# Grey-Theory-Based Optimization Model of Emergency Logistics Considering Time Uncertainty

**DOI:** 10.1371/journal.pone.0139132

**Published:** 2015-09-29

**Authors:** Bao-Jian Qiu, Jiang-Hua Zhang, Yuan-Tao Qi, Yang Liu

**Affiliations:** 1 School of Mathematical Sciences, University of Jinan, Jinan, Shandong China; 2 School of Management, Shandong University, Jinan, Shandong China; Nankai University, CHINA

## Abstract

Natural disasters occur frequently in recent years, causing huge casualties and property losses. Nowadays, people pay more and more attention to the emergency logistics problems. This paper studies the emergency logistics problem with multi-center, multi-commodity, and single-affected-point. Considering that the path near the disaster point may be damaged, the information of the state of the paths is not complete, and the travel time is uncertainty, we establish the nonlinear programming model that objective function is the maximization of time-satisfaction degree. To overcome these drawbacks: the incomplete information and uncertain time, this paper firstly evaluates the multiple roads of transportation network based on grey theory and selects the reliable and optimal path. Then simplify the original model under the scenario that the vehicle only follows the optimal path from the emergency logistics center to the affected point, and use Lingo software to solve it. The numerical experiments are presented to show the feasibility and effectiveness of the proposed method.

## Introduction

In recent years, a number of disasters happen occasionally, such as the tsunami in Southeast Asia at the end of 2004, Hurricane Katrina in U.S. in 2005, Wenchuan earthquake in 2008, the super earthquake in Philippine in March 2012. All these disasters caused huge casualties and property losses to people. Therefore, emergency logistics play an important role in disaster relief operations. Emergency Logistics is a special logistics activity that provides emergency supplies for unexpected natural disasters, public health emergencies and other unexpected incidents, and aims at maximizing the time benefit and minimizing the loss of the disaster. And many researchers have focused on this subject since the late 1980s.

Haghani and Oh [1] present a large-scale multi-commodity multi- modal network flow model with time windows in the context of disaster relief operations. Two different heuristics are proposed for the solution of the problem. Vitoriano et al. [2] proposed a multi-criteria optimization model for aid distribution, and establish a decision support system. Ozdamar et al. [3] present a network-based multi-period model for emergency logistics. Tzeng et al. [4] develop a multi-objective relief-distribution model for designing real-life relief delivery systems. Their model features three objectives, including minimization of the total cost, minimization of the total travel time, and maximization of the minimal satisfaction during the planning period. Yan and Shih [5] propose multi-objective, multi-commodity network model in order to minimize the time of road maintenance and material distribution. Nolz et al. [6] develop a multi-objective model for emergency logistics. This model encompasses three objective functions, including minimizing the risk, maximizing the coverage provided by the logistics system and minimizing the total travel time. Najafi et al. [7] propose a multi-objective, multi-mode, multi-commodity, and multi-period stochastic model to manage the logistics of both commodities and injured people in the earthquake response, and develop a robust approach to make sure that the distribution plan performs well under the various situations. Barbarosoglu and Arda [8] present a two-stage stochastic programming model for relief distribution that includes uncertain arc capacities and demands to represent the lack of knowledge following a disaster. Ali Bozorgi-Amiri et al. [9] propose the multi-objective stochastic programming model of relief logistics with uncertainty. Sheu [10] presents a hybrid fuzzy clustering-optimization approach to coordinate the relief logistics flows in a three-layer relief supply network during the crucial rescue period. Raktim Pal and Indranil Bose [11] propose mixed integer programming model for deployment of roadway incident response vehicles with reliability constraint, and divide disaster points into big and small ones which consider different sizes of vehicles. Zhang et al. [12] propose an integer mathematical model to allocate the available resources to demand points subject to constraints on multiple resources and possible secondary disasters. Yuan and Wang [13] construct a model to express the effect that the disaster extension influenced the travel speed. Chang et al. [14] propose a greedy-search-based, multi-objective, genetic algorithm capable of regulating the distribution of available resources and automatically generating a variety of feasible emergency logistics schedules for decision-makers.

From the above literature, the existing models are mainly divided into two categories: deterministic and undeterministic model; and the objective functions are mainly minimization of the total cost, minimization of the total travel time, or minimizing the risk; and uncertainty factors include demand, and the number of vehicles etc. While the literature considering the incomplete information and uncertain time is rare. Sheu [15] presents a dynamic relief-demand management model for emergency logistics operations under imperfect information conditions in large-scale natural disasters. Their model consists of three main steps: data fusion to forecast relief demand in multiple areas, fuzzy clustering to classify affected area into groups, and multi-criteria decision making to rank the order of priority of groups. Zhang et al. [16] study route selection of emergency logistics which the travel time is not only related with the length of the arc, but it is also related with the travel speed of arc since the travel speed is a decreased function respect to distance. In fact, when disaster happens, the information of the state of the roads is not complete, because it is unexpected and the rescue time is urgent. Based on this consideration, this paper will assess the multiple roads of transportation network based on grey theory and select the reliable and optimal path, during which the vehicle only follows the optimal path from the emergency logistics center to the affected point. In addition, the travel time is uncertainty, and we have known that the travel time will go up generally with the increase of vehicles on the path. Therefore, this paper will consider the situation that the travel time is uncertainty, study the emergency logistics problem of multi-center, multi-commodity, and single-affected-point based on grey theory, and establish the nonlinear programming model that objective function is the maximization of time-satisfaction degree.

The section 2 describes some basic assumptions and symbols. The section 3 first establishes the relationship between travel time *t*_*i*_ and traffic volume *v*_*i*,_, defines time-satisfaction degree function, and establishes a nonlinear programming model. The section 4 describes how to determine the optimal path based on grey theory and then simplifies the above model. Finally, an illustrative example and conclusion are presented in Sections 5 and 6 respectively.

## Problem Description

There exist *m* emergency logistics centers that can totally provide *n* types of emergency supplies to the affected point. There is 1 affected point, when the affected point suffers disaster; emergency logistics center will provide appropriate relief services for the affected point. The loading capacity of vehicles is equal.

This paper will establish the nonlinear programming model that objective function is the maximization of time-satisfaction degree, and select optimal path based on grey theory and schedule emergency supplies from multi-emergency logistics center to single affected point.

### 2.1 Assumption

1. demand for various emergency supplies of affected point is constant, and the supplies must be delivered within a certain time.
2. all emergency logistics center are capable of satisfying the demand for various emergency supplies of the affected point, and the vehicle is allowed to a non-loaded transport.
3. every emergency logistics center has only one type of vehicle, and the capacity of vehicle is less than the demand of the affected point for various emergency supplies, the vehicles of each center are certain, and the total vehicle capacity of all the emergency center is greater than the total necessary emergency supplies of affected point.
4. paths from different emergency logistics centers to the affected point are in no coincidence, and different paths from the same center to affected point can coincide.
5. Volume-delay functions [17,18,19] are used to express the travel time on a road link as a function of the traffic volume *v*. The most widely used Volume-delay functions are the Bureau of Public Roads (BPR) [18,19], which are defined as $t_{}^{BPR}(v)=t_{}^{0}[1+{\left(\frac{v}{c}\right)}^{\alpha }],\;\alpha >1,$ where *c* is the capacity of the road and *v* is the traffic volume of the road. In this paper, for the purpose of processing convenience, we let *α* = 2.
6. all vehicles will set off to the affected point together after being loaded.

### 2.2 Symbols Illustration

*i*, *j*, *k*, *l* respectively represents emergency logistics center, emergency supplies, vehicles, sequence numbers of paths;

*F*, *f*, *q* respectively denote the fixed cost of using a vehicle, the cost per unit distance and the loading capacity of the vehicle;

*A* denotes budget; *T* represents the latest allowed arrival time to the affected point estimated by experts;

*k*_*i*_、*l*_*i*_ respectively denotes the number of the vehicles owned by center *i* and the number of paths to the affected point;

*ρ*_*j*_ *、P*_*j*_ respectively denotes the weight of unit material and the demand for material *j* of the affected point;

*D*_*ij*_ represents the amount of material *j* that center *i* can provide;

*t*_*i*_^*l*^、$y_{ik}^{l}$ respectively represents the actual time from center *i* to the affected point via the *lth* route and the time used normally;

*d*_*i*_^*l*^、*Q*_*i*_^*l*^ respectively represents the distance of the *lth* path from center *i* to the affected point and the road loading capacity;

***y***_***ik***_ = 0 or 1, when the *kth* vehicle is called of center *i y*_*ik*_ = 1, otherwise *y*_*ik*_ = 0,

$y_{ik}^{l}=0$ or 1, when the *kth* vehicle of center *i* follows the *lth* path $y_{ik}^{l}=1$, otherwise $y_{ik}^{l}=0$,

*x*_*ijk*_ indicates the amount of material *j* loaded by the *kth* vehicle of center *i*;

Note: The range of the symbols above *i*, *j*, *k*, *l* is respectively *i* = 1, 2, …, *m*, *j* = 1,2, …, *n*, *k* = 1, 2,…, *k*_*i*_, *l* = 1,2, …, *l*_*i*_ without other special instructions.

## The Model Establishment

### 3.1 The Relationship between the Travel Time *t*_*i*_ and the Traffic Volume *v*_*i*_ of the Path

With the increase of the vehicles on path, the vehicle speed will be reduced, and increase with travel time correspondingly. Based on the assumption 5, the relationship between the actual time and the traffic volume of the *lth* route from center *i* to the affected point is as follows:

$$t_{i}^{l}=t_{il}^{0}[1+{\left(\frac{\sum_{k=1}^{k_{i}}y_{ik}^{l}}{Q_{i}^{l}/q}\right)}^{2}]$$ (1)

Here, it is allowed that the actual traffic volume exceeds the capacity of the path, thus it comes at the cost of increasing the travel time on the path. We adjust the number of vehicles on each path by maximizing time-satisfaction degree, making that it not exceeds the capacity of path too much, so in the model later we do not consider the capacity limitation of each optimal path, *i*.*e*.

$$\sum_{k=1}^{k_{i}}\sum_{j=1}^{m}x_{ijk}y_{ik}^{l}\leq Q_{i}^{l},\;i=1,2,..,m,l=1,2,..,l_{i}$$ (2)

### 3.2 The Time-Satisfaction Degree Function

**Fig 1:**
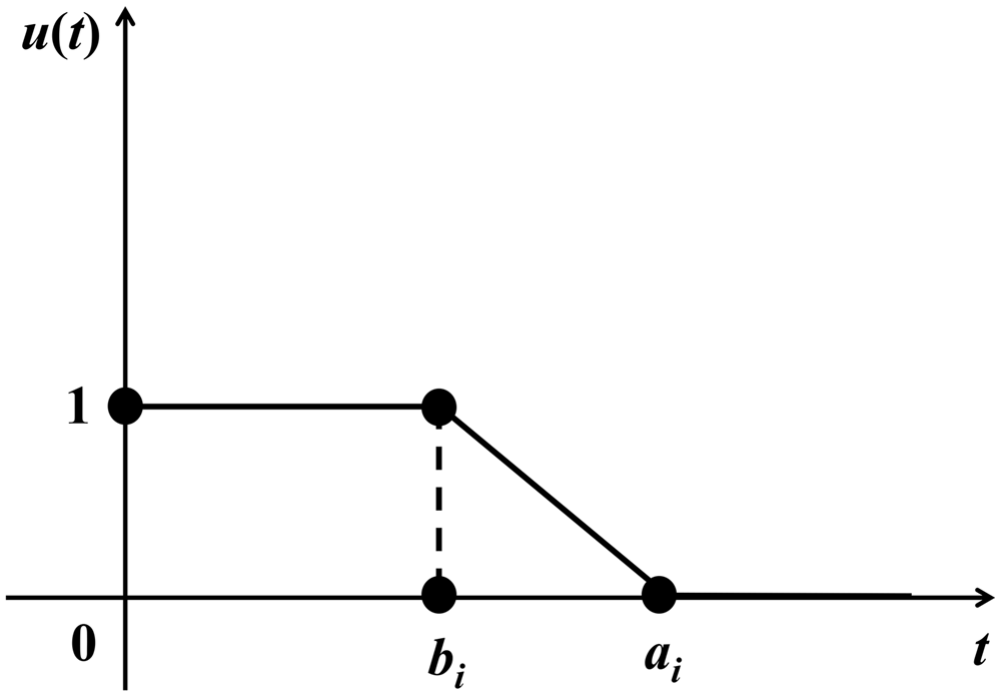
The time-satisfaction function.

Because of the uncertainties on the number of vehicle and the distribution plan of emergency supplies, the arrival time of vehicles is also uncertain. The longer the travel time used to transport the emergency supplies, the lower satisfaction degree of the affected point, on the contrary, the higher satisfaction degree. Define the time-satisfaction degree function as shown in Fig 1.

That is

$$u_{i}(t)=\{\begin{array}{c} \;1,\text{ 0}<t<b_{i}\\ \frac{a_{i}-t}{a_{i}-b_{i}},\;b_{i}\leq t\leq a_{i}\\ \;0,\;t>a_{i} \end{array}$$ (3)

### 3.3 The Model of the Problem

$$\text{max}\sum_{i=1}^{m}\sum_{l=1}^{l_{i}}u_{i}(t_{i}^{l})$$ (4)

$$s.t.\;t_{i}^{l}=t_{il}^{0}[1+{\left(\frac{\sum_{k=1}^{k_{i}}qy_{ik}^{l}}{Q_{i}^{l}}\right)}^{2}],\;i=1,2,..,m,\;l=1,2,..,l_{i}$$ (5)

$$t_{i}^{l}\leq T,\;i=1,2,..,m,\;l=1,2,..,l_{i}$$ (6)

$$\;\sum_{i=1}^{m}\sum_{k=1}^{k_{i}}\sum_{l=1}^{l_{i}}\left(F+f\cdot d_{i}^{l}\right)y_{ik}^{l}\leq A$$ (7)

$$\sum_{j=1}^{n}\rho_{j}x_{ijk}\leq qy_{ik},\;i=1,2,..,m,\;k=1,2,..,k_{i}$$ (8)

$$\sum_{k=1}^{k_{i}}x_{ijk}\leq D_{ij},\;i=1,2,..,m,\;j=1,2,..,n$$ (9)

$$\sum_{i=1}^{m}\sum_{k=1}^{k_{i}}x_{ijk}\geq P_{j},\;j=1,2,..,n$$ (10)

$$\;y_{ik}=\sum_{l=1}^{l_{i}}y_{ik}^{l},\;i=1,2,..,m,\;k=1,2,..,k_{i}$$ (11)

$$y_{ik}=0\;or\;1,\;y_{ik}^{l}=0\;or\;1,\;i=1,2,..,m,\;k=1,2,..,k_{i},\;l=1,2,..,l_{i}$$ (12)

$$x_{ijk}\text{ are non−negative integer},\;i=1,2,..,m,\;j=1,2,..,n,\;k=1,2,..,k_{i}$$ (13)

Formula (4) is the objective function, which denotes that the sum of time-satisfaction degree from all emergency centers to the affected point; Eqs (5) and (6) represents the travel time from center *i* to affected point via the *l-th* route does not exceed the preset time; Eq (7) shows that the total cost does not exceed the budget; Eq (8) represents the capacity limitations of the vehicle; Eq (9) denotes the capacity constraints of material *j* that center *i* can provide; Eq (10) represents the total demand for material *j* in order to satisfy the affected point; Eq (11) represents that the *k*-th vehicle in center *i* can at most choose one path to transport the materials to the affected point. Eq (12) represents 0–1 variables; Eq (13) represents non-negative integer constraints.

## The Optimal Path Determination

### 4.1 Prepare Knowledge

#### (1) Index system for the optimal path evaluation

When selecting the optimal path in emergency logistics, we need establish the selection and evaluation index system. In this paper, we consider the six indicators: the safety of transportation (ST), convenience of transportation organization (CTO), congestion level of path (CLP), the difficulty of traveling (DT), path information (PI), and path conditions (PC).

#### (2) Grey class, grey number and whitenization weight function [20]

In the study of system, due to different disturbance and limitation of cognition level, people get some uncertain information. The grey system theory is a method dealing with systems with few data, poor information, and uncertainty question. The number that we only know the range of its value but don't know the exact value is called the grey number. Whitenization weight function is used to describe the preference degree of a grey number for different value of its value range.

The normal whitenization weight function *f*_1_ (*x*) is described as:

$$f_{1}(x)=\{\begin{array}{cc} L(x), & x\in [x_{1},x_{2}]\\ 1, & x\in [x_{3},x_{4}]\\ R(x), & x\in [x_{5},x_{6}], \end{array}\;$$ (14)

where *a1*<*a2*, *f1(x)* is a continuous function, *L(x)* is a non-decreasing function, and *R(x)* is a non-increasing function. In general, *L*(*x*) = (*x*-*x*_1_)/(*x*_2_-*x*_1_), *R*(*x*) = (*x*_6_-*x*)/(*x*_6_-*x*_5_).

**Fig 2:**
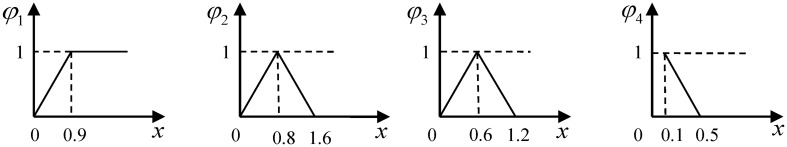
The whitenization weight functions of four grey classes.

For path selection problem, this paper divides the grey class into four classes: excellent, good, medium and poor. According to the importance of each grey class, we preset the grey class weight *c* = (1, 0.8, 0.6, 0.4). The whitenization weight function of the first grey class (excellent) is *φ*_1_, the whitenization weight function of the second grey class (good) *φ*_2_, the whitenization weight function of the third grey class (medium) *φ*_3_, the whitenization weight function of the fourth grey class (poor) *φ*_4_, are respectively shown in Fig 2:

These functions are as following:

$$\begin{array}{l} \varphi_{1}(x)=\{\begin{array}{cc} \frac{x}{0.9} & x\in [0,0.9]\\ 1, & x\in [0.9,+\infty ] \end{array},\;\varphi_{2}(x)=\{\begin{array}{cc} \frac{x}{0.8} & x\in [0,0.8]\\ \frac{1.6-x}{1.6-0.8}, & x\in [0.8,1.6] \end{array}\\ \varphi_{3}(x)=\{\begin{array}{cc} \frac{x}{0.6} & x\in [0,0.6]\\ \frac{1.2-x}{1.2-0.6}, & x\in [0.6,1.2] \end{array},\;\varphi_{4}(x)=\{\begin{array}{cc} 1 & x\in [0,0.1]\\ \frac{0.5-x}{0.5-0.1}, & x\in [0.1,0.5] \end{array} \end{array}$$ (15)

#### (3) Determine the weight of evaluation index

The weight vector *w* of evaluation index is determined as follows: first, obtain the judgments of various experts in the form of questionnaire. Each expert writes 1 on the index that he considers the most important, and writes 2 on the second important… writes *M* on the least important.

After attaining the data, process the obtained data as follows

$$w_{i}=\frac{2[N(1+M)-R_{i}]}{MN(1+M)}$$ (16)

Wherein, *w*_*i*_ represents the weight of the *i-th* index; *M* represents the number of index; *N* represents the number of experts; *R*_*i*_ represents the sum of the assessment on the *i-th* index by various experts.

### 4.2 Selection of The Optimal Path Based on Grey Theory

#### (1) Symbols illustration

*i* represents index number, *i* = 1,2, …, 6; *j* represents the evaluation grey class number, *j* = 1,2,3,4;

*g*_*si*_ denotes the *i-*th evaluation index of the *s-th*(*s* = 1,2,…, l) path;

*β* = (*β*_*si*_)_l×6_ represents the evaluation information matrix, wherein *β*_*si*_ is attained from the normalization processing of *g*_*si*_.

*X* ^(s)^ = (*x*_*ij*_^(s)^)_6×4_, where *x*_*ij*_^(s)^ represents the grey evaluation coefficient on evaluation index *i* belongs to the *j-th* grey class of the *s-th* path;

*P* ^(s)^ = (*p*_*ij*_^(s)^) _6×4_, wherein, *p*_*ij*_^(s)^ represents the grey evaluation weight on evaluation index *i* belongs to the *j-th* grey class of the *s-th* path;

*y* ^(s)^ represents the vector of synthetic evaluation results on the *s-th* path;

*Y* represents synthetic situation matrix of the path;

*z* represents the synthetic evaluation vector of the path.

#### (2) The steps to determine the optimal path based on grey theory

The steps are as follows.

Step 1: determine the index value for each path and normalize to obtain path evaluation information matrix *β*

Based on the actual situation, determine each index value for each path, since the unit and standards of each index value are different, the data has to be normalized. If the value of evaluation index is the bigger the better, then process according to the formula $\beta_{si}=g_{si}/\underset{i}{\text{max}}g_{si}$; otherwise process it according to the formula $\beta_{si}=g_{si}/\underset{i}{\min}g_{si}$. After processing, we can obtain the information matrix of path *β* = (*β*_*si*_)_*n*×*m*_.

Step 2: Calculate the grey evaluation weight matrix for each path

Seek the grey evaluation coefficient matrix *X* ^(s)^ = (*x*_*ij*_^(s)^)_6×4_ belongs to each evaluation grey class of each index value on each path, wherein *x*_*ij*_^(s)^ = *φ*_*j*_(*β*_*si*_), then seek the grey evaluation weight matrix *P* ^(s)^ = (*p*_*ij*_^(s)^)_6×4_ to each evaluation grey class *j* of each index on the *s-th* path.

Step 3: Calculate the synthetic evaluation value matrix *Y* of each path

$$Y=\left[\begin{array}{l} y^{\left(1\right)}\\ y^{\left(2\right)}\\ ...\\ y^{\left(l\right)} \end{array}\right]=\left[\begin{array}{l} y_{1}^{\left(1\right)}\;y_{2}^{\left(1\right)}\;y_{3}^{\left(1\right)}\;y_{4}^{\left(1\right)}\\ y_{1}^{\left(2\right)}\;y_{2}^{\left(2\right)}\;y_{3}^{\left(2\right)}\;y_{4}^{\left(2\right)}\\ ...\;...\;...\;...\\ y_{1}^{\left(l\right)}\;y_{2}^{\left(l\right)}\;y_{3}^{\left(l\right)}\;y_{4}^{\left(l\right)} \end{array}\right]$$ (17)

Wherein*y* ^(*s*)^ = *wP* ^(*s*)^, which represents the synthetic evaluation of each grey class on the *s-th* path.

Step 4: Determine the optimal path

Seek the synthetic evaluation value for each path *z = Yc* = (*z*_1_, *z*_2_, *…*, *z*_*l*_)^*T*^, where *z*_*s*_, *s* = 1,2,…, *l* is the synthetic evaluation value of the *s-th* optional path, and the path with the maximum synthetic evaluation value is the optimal path.

### 4.3 Simplify the Original Model

After obtaining the optimal path from each center to the affected point, we limit that it only follows the optimal path from emergency logistics center *i* to affected point. Under this assumption, if the vehicle of center *i* is called, it can only take the optimal path, then the 0–1 variable $y_{ik}^{l}$ in the model can be removed. The symbols $t_{i}^{l},t_{il}^{0},y_{ik}^{l},Q_{i}^{l},d_{i}^{l}$ will become the corresponding information on the optimal path from center *i* to the affected point, which are respectively represented by *t*_*i*_、*t*_*i*_^0^、*y*_*ik*_、*Q*_*i*_、*d*_*i*_. At this point, this simplified model is obtained as follows:

$$\text{max}\sum_{i=1}^{m}u_{i}(t_{i})$$ (18)

$$s.t.\;t_{i}=t_{i}^{0}[1+{\left(\frac{\sum_{k=1}^{k_{i}}qy_{ik}}{Q_{i}}\right)}^{2}],\;i=1,2,..,m$$ (19)

$$t_{i}\leq T,\;i=1,2,..,m$$ (20)

$$\sum_{i=1}^{m}\sum_{k=1}^{k_{i}}(F+f\cdot d_{i})y_{ik}\leq A$$ (21)

Eqs (8), (9), (10) and (13)

$$y_{ik}=0\;or\;1,\;i=1,2,..,m,\;k=1,2,..,k_{i}$$ (22)

There is piecewise function in the objective function of the above model, and the model is a nonlinear programming model. This paper will use the software lingo to solve the optimization model.

## Numerical Experiments

In this paper, we use an example of the earthquake to analyze in a certain area. Assume that each emergency logistics center can obtain some information after the disaster. Given that the affected points need 6 types of emergency supplies which are tents, quilts, drinking water, bread, bandages and drug(denoted respectively by *j* = 1,2,3,4,5,6), the unit weight (kg) of 6 materials is 80, 5, 24, 20, 15,30 respectively. There are 5 emergency logistics centers (denoted respectively by *i* = 1,2,3,4,5) near the affected points which can provide emergency supplies. Each center has 4 vehicles, and the vehicle loading capacity is *q* = 50(ton), the fixed cost of using a car is *F* = 5000(Yuan), the average cost for driving 100 kilometers is *f* = 120(Yuan), the budget of transportation expenses is *A* = 1,000,000 (Yuan). Experts estimate that the latest rescue time is *T* = 24(hour), and we set *a*_*i*_ = 24, *b*_*i*_ = 12. The other relevant data is in Table 1 and Table 2.

**Table 1:** Data information of each path from emergency logistics center to the affected points.

Center No.	Path No.	ST	CTO	CLP	DT	PI	PC	*Q*_*i*_(ton)	*d*_*i*_(km)	*t*_*i*_^0^(h)
1	1	1	0.83	0.53	0.36	0.50	0.88	200	120	4
	2	0.85	0.78	0.70	0.30	0.40	1	160	210	7
	3	0.60	0.70	0.48	1	0.60	0.70	140	300	10
	4	0.88	0.70	0.75	0.40	0.55	0.75	180	120	4
2	5	1	0.80	0.40	0.50	0.60	0.70	160	240	8
	6	0.80	0.70	0.70	0.66	0.70	0.70	200	240	8
3	7	0.88	0.87	0.60	0.60	0.80	0.60	120	150	5
	8	0.90	0.88	0.40	0.50	0.70	1	160	150	5
	9	0.81	0.81	0.55	0.7	0.6	0.88	140	120	4
4	10	0.8	0.75	0.7	0.75	0.8	1	200	210	7
	11	0.7	1	0.6	0.76	0.7	0.7	180	168	5.6
	12	1	0.8	0.6	0.55	0.7	0.9	190	240	8
5	13	1	0.75	0.55	0.37	0.60	0.88	130	138	4.6
	14	0.88	0.65	0.60	0.7	0.70	0.76	140	210	7
	15	0.65	0.60	0.65	0.53	0.60	0.85	120	180	6
	16	0.78	0.70	0.75	0.40	0.55	0.75	150	198	6.6

**Table 2:** Inventory information of emergency logistics center and demand information of affected points.

Center No.	Tents	Quilts	Drinking water	Bread	Bandages	Drugs
1	3000	8900	2280	1600	480	900
2	1000	6000	1300	800	0	0
3	1500	2500	2620	1600	370	800
4	0	2840	1920	700	300	1500
5	1000	0	510	1800	420	1000
Demand *P*_*j*_	4800	18000	6500	5000	900	2000

### 5.1 Select the Optimal Path Based on Grey Theory

According to steps in section 4.2, we can obtain the optimal path from each emergency logistics center to the affected points easily. The final result is shown in Table 3.

**Table 3:** The comprehensive evaluation value and the 0ptimal path of each center.

Center No.	Path No.	Comprehensive evaluation value	Optimal path
1	1	0.886903	
	2	0.874953	
	3	0.863137	
	4	0.895798	√
2	5	0.853614	
	6	0.863061	√
3	7	0.854946	
	8	0.86037	√
	9	0.855805	
4	10	0.852662	√
	11	0.818108	
	12	0.78024	
5	13	0.862939	
	14	0.869683	
	15	0.847509	
	16	0.905524	√

From the Table 3, we know that center 1 chooses the 4th path, center 2 chooses the 6th path, center 3 chooses the 8th path, center 4 chooses the 10th path, and center 5 chooses the 16th path.

### 5.2 Solve the Optimal Solution of The Model

Using lingo software to solve it, we get that the maximum time-satisfaction degree is 4.790625, and the optimal distribution scheme is shown in Table 4, and the arrival time from emergency logistics center to the affected points is shown in Table 5.

**Table 4:** The optimal material distribution scheme.

Center No.	Vehicle No.	Tents	Quilts	Drinking water	Bread	Bandages	Drugs
1	1	13	8769	213	0	0	0
	2	37	0	339	1585	479	0
	3	625	0	0	0	0	0
	4	625	0	0	0	0	0
2	1	0	6000	121	800	0	0
	2	625	0	0	0	0	0
	4	375	0	833	0	0	0
3	1	0	332	1951	75	0	0
	2	252	32	612	43	0	471
	3	625	0	0	0	0	0
	4	623	27	1	0	0	0
4	1	0	2840	0	697	300	576
	2	0	0	991	0	0	1
	4	0	0	929	0	0	923
5	2	461	0	0	0	0	27
	3	365	0	510	0	121	0
	4	174	0	0	1800	0	2

**Table 5:** The arrival time from emergency logistics center to the affected points(h).

Center No.	1	2	3	4	5
Arrival time	8.938272	12.5	12.8125	10.9375	13.2

### 5.3 Further Discussion

According to the assumption 5 in section 2.1, we supposed that travel time of the path is quadratic function of the traffic volume. It can be shown in Table 6 that the running time of global optimal solution will become very long when the number of vehicles increases at every logistics center, which can't be accepted in the actual problem. So when the running time is too long, we generally take the local optimal value as satisfactory solution of the practical problems. However, under the assumption that travel time of the path is linear function of the traffic volume, the growth speed of the running time of global optimal solution is not fast when the number of vehicles increases at every logistics center. And besides, the running time becomes shorter when the number of vehicles increases to a certain value, because the objectives function value can reach the maximum value 5 soon.

**Table 6:** The comparison of the results under different assumptions.

	vehicle number	5×4	5×5	5×6	5×7	5×8
Nonlinear	Optimal value	4.790625(Global)	4.263889(Local)	4.566081 (Local)		
	Operation time (hh:mm:ss)	00:05:04	00:30:58	01:10:15		
Linear	Global optimal value	4.7125	4.911458	4.979167	5	5
	Operation time(mm:ss)	02:29	04:37	04:43	02:01	02:00

Therefore, when the number of vehicles is small, a quadratic relationship setting can get the optimal solution in a short time. Otherwise, a linear relationship is better.

## Conclusions

Emergency logistics problem is a hot topic in the field of management science, and there are a lot of issues which are worth studying. This paper focuses on the emergency logistics problem with multi-center, multi-commodity, and single-affected-point. According to the actual conditions that the path near the disaster point may be damaged, the information of the state of the paths is not complete, and the travel time is uncertainty, we propose the nonlinear programming model whose objective is maximizing the time-satisfaction degree. We obtain the optimal paths from each emergency logistics center to the disaster point based on grey theory, and then simplify the original model considering that the vehicle only follows the optimal path from the emergency logistics center to the affected point, and finally use Lingo software to solve it. The results of numerical experiment show the feasibility and effectiveness of the proposed method.

In the future, we will study effective approximation algorithms or heuristic algorithms to solve the simplified nonlinear programming model. And we’ll study the application of this method based on the actual case, such as “WenChuan” earthquake. These can help the relevant government departments develop the emergency plans scientifically and effectively, and perform the rescue quickly and efficiently.

## Supporting Information

S1 Table Data information of each path from emergency logistics center to the affected points. The meaning of the abbreviations are as follows, ST: safety of transportation, CTO: the convenience of transportation organization, CLP: the congestion level of path, DT: the difficulty of traveling, PI: path information, PC: path conditions.
(XLSX)
